# Ethical considerations in the use of GPS-based movement tracking in health research – lessons from a care-seeking study in rural west India

**DOI:** 10.7189/jogh.09.010323

**Published:** 2019-06

**Authors:** Aditi Apte, Vijendra Ingole, Pallavi Lele, Andrew Marsh, Tathagata Bhattacharjee, Siddhivinayak Hirve, Harry Campbell, Harish Nair, Sarah Chan, Sanjay Juvekar

**Affiliations:** 1 KEM Hospital Research Centre (KEMHRC), Vadu Rural Health Program, India; 2 ISGlobal, Barcelona, Spain; 3 Institute for International Programs, Johns Hopkins University Bloomberg School of Public Health, Baltimore, Maryland, USA; 4 INDEPTH Network, East Legon, Accra, Ghana; 5 Usher Institute of Population Health Sciences and Informatics, University of Edinburgh, Edinburg, Scotland, UK

Geospatial technologies (GSTs) refer to a range of modern tools used for geographical mapping and analysis of earth’s features, which include Global Positioning System (GPS), Geographical Information System (GIS) and Remote Sensing [1]. Miniaturization of GPS devices has enabled their integration into mobile phones, wearables and vehicles and thus their use as part of “mHealth” [2]. The GPS is a versatile technology that can be used essentially to monitor any outdoor activity, and is currently used for a wide range of purposes including transport, navigation, law enforcement, scientific research and leisure activities [3].

GSTs have been used in the surveillance and monitoring of infectious diseases, assessments of environmental health risk, and the analysis of disease policy and planning. GSTs help health programmes to map geographical access to public health resources, community transport services, or association of health events with their environment [4-6]. GST has been used in Peru to track mobility patterns related to the risk of transmission of Dengue [7]; in South Africa to trace patients receiving treatment for tuberculosis [8]; and for mapping of Kala Azar, lymphatic filariasis and Kyasanur forest disease in coastal regions of India [9,10]. GSTs have been used to monitor movement patterns of humans related to their health behaviour. GPS devices have been used to track movement of cognitively impaired individuals such as elderly people suffering from dementia or Alzheimer disease in order to prevent wandering [11]. They are used to measure outdoor walking capacity of patients suffering from peripheral artery disease, which can help to determine severity of disease in these patients [12]. Health researchers have combined GPS with activity monitoring to measure exercise or sedentary behaviour of people [13].

Ascertainment of care-seeking is important in public health management in order to plan national health programs and assessing quality of health care. Nguyen et al. demonstrated use of GPS-based devices for ascertainment of hospitalisations [14]. Paz-Soldan et al. compared use of GPS-based devices to obtain fine-scale human mobility data and compared it with semi-structured interviews [15].

GSTs have provided health administrators and researchers with unprecedented access to personal information regarding an individual’s activities, movements at home and in the community. This enables health information to be linked to spatio-temporal data in new ways and in much greater detail, opening up possibilities for innovative research and insights into health-related behavior. At the same time, however, this new capacity for surveillance has raised issues related to privacy, confidentiality, access to information, and the possibility of misuse of location data in health research. These and other issues manifest in particular ways when these technologies are applied for research in the context of developing countries [16,17]. While there is an emerging body of literature exploring the ethical considerations of location tracking, few studies address these through the lens of scientific research and fewer still do so in the context of low- and middle-income countries [2,4-6,14].

This commentary aims to discuss ethical issues related to the use of GPS-based movement tracking during a care-seeking study in rural India.

## INVESTIGATING CARE-SEEKING FOR CHILDHOOD ILLNESSES USING LOCATION DATA: A CASE STUDY

TrackCare, a location-aware smartphone application, was used to track care-seeking behaviour of mothers of under-five children for common childhood illnesses in a study conducted in 22 villages of Vadu HDSS, Pune, Maharashtra [18]. The study consisted of phone groups and non-phone groups and compared the potential care-seeking events identified through TrackCare, a smartphone application with care-seeking events identified through participant interviews. Consenting mothers in the phone group were given a dual SIM phone which they were asked to carry with them whenever any care-seeking event took place. They were followed up monthly for a period of six months.

TrackCare was an Android-based application designed for this study to track the phone’s location each minute and transfer these data to a central server each hour [19]. This information was transmitted hourly to a central study server after which the stored data from the device were deleted. When hourly transfer was not possible due to poor connectivity, cumulated data were transferred during the next scheduled transfer. TrackCare was designed to function continuously and automatically once the device was turned on and was password-protected from uninstallation preventing its removal except by study staff. The movement data generated by phones were stored on secured servers in University of Edinburgh and Vadu HDSS.

The study was reviewed and approved by institutional ethics committees of KEM Hospital Research Centre, Pune and the Centre for Population Health Sciences at the University of Edinburgh.

**Figure 1:**
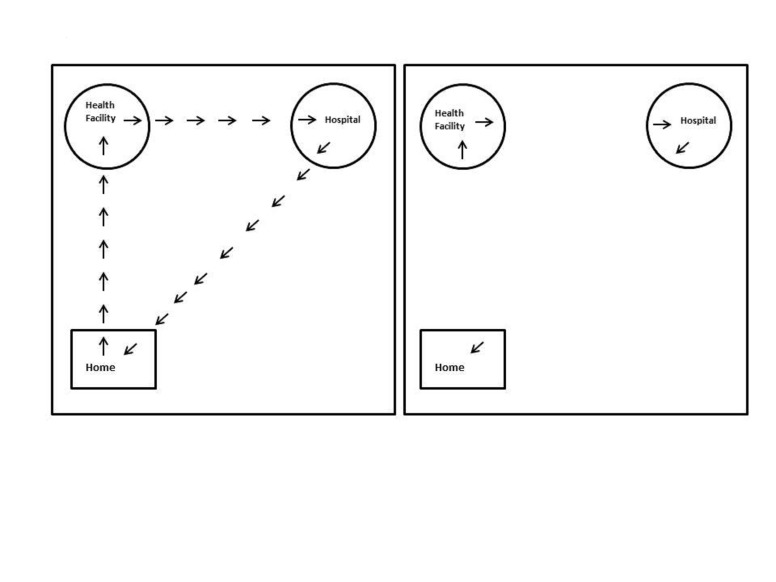
Pictorial depiction of data collection and data storage in informed consent of care-seeking study. Left – geospatial data collected in GPS devices during the study. Right – Data that were archived after the study completion.

All the study participants were administered a written informed consent in local language (Marathi) after explaining the nature of the study procedures along with risks and benefits associated with study participation. The informed consent document was designed using simple language and covered different aspects of the study including purpose of the study, voluntary nature of participation, type of data shared, confidentiality of the data, risks and benefits of the study. The participants who received a phone could use it for their personal use as well using a second SIM card. There was no compensation paid for participation in this study, however the participants in the phone group could retain the smartphones at the end of the study. The consent form had a pictorial depiction of the geospatial data archival process explaining that only data for care-seeking visits were retained during archival and all other location data were deleted (**Figure 1**).

## POTENTIAL ETHICAL CHALLENGES IN USING GPS IN HEALTH AND RESEARCH: LEARNINGS FROM CARE-SEEKING STUDY

The ability to use GPS to trace the movements of people raises important ethical issues [3]. Geospatial technology for personal location tracking presents a global concern and there is geo-location privacy legislation in place in some countries which prohibits use of this technology for routine surveillance activities [20]. In India, the Geospatial Information Regulation Bill (GIRB) has been drafted by Government and is under review, which mandates prior permission to use geospatial data from government licensing authority [21].

As our experiences during the present care-seeking study illustrate, these issues also present challenges for the use of GPS as a health research tool in ways that must be taken into account when designing and conducting research, especially in low and middle-income countries.

### Privacy and confidentiality

The primary concern in relation to the use of GPS is the amount of information that can be deduced from the person’s movements and how that information may be used [16]. In the first place, continuous monitoring of one’s activity by a researcher, even where consent is initially given, poses the threat of invasion of privacy and may lead to psychological implications from the feeling of being “watched” [17]. Bearing in mind the privacy implications, only movement data for visiting health facilities from the care-seeking study were archived, avoiding archival of other movement data as agreed during the consent. The location data available to researchers were de-identified and included geo-coordinates of households and health facilities only. Any data that included personal identifiers were first transferred to electronic database stored under high security. The data were then de-identified before analysis. The phone data were identified by numeric ID assigned to each phone and the participant was identified by study ID; identifiable data were encrypted before storage on servers. This strategy prevented access to unnecessary movement data by researchers as this may have ethical implications. Field research assistants were trained to maintain necessary confidentiality while handling probable visit data.

**Figure:**
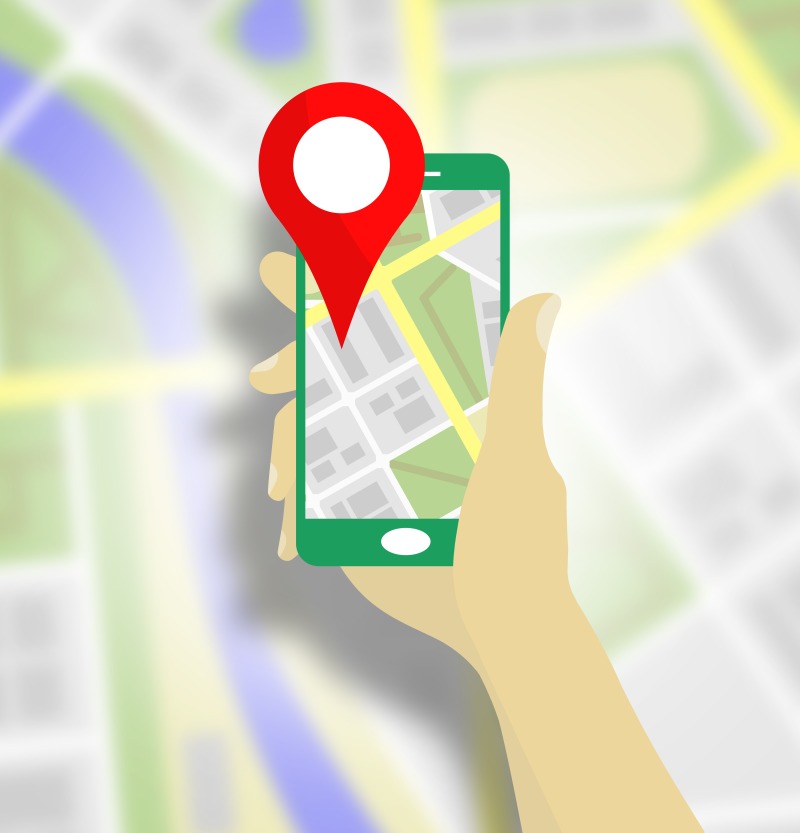
Photo: GPS navigation (from: https://pixabay.com/illustrations/navigation-gps-location-google-2049643/)

While conducting the care-seeking study, we found that the overall awareness regarding the nature of GPS technology was low amongst the rural population. However, concerns over confidentiality were raised by several study participants some even asking whether the device could record conversations or videos of their activities. There was one refusal to consent based on the issue of confidentiality.

### Spatial re-identification risk and negligence in publication

Based on the location history of the device, it is possible to identify the location of a particular individual and trace his or her movement with reasonable accuracy. From the observed movements, these data may then be associated with a specific individual, meaning that de-identification is effectively impossible [10]. The limited scope for anonymisation of movement data is a big challenge in global health research for sharing of data. Accidental self-disclosure leading to breaches of locational privacy is a possibility: if participants are given access to GST-enabled smart phones with which they are unfamiliar, their incidental use of the devices may result in inadvertently revealing information about themselves. In care-seeking study, first, the electronic data were anonymised to protect identity of an individual; second, geospatial coordinates of only health facilities were enabled avoiding revealing of other movement data.

There are examples in literature where the exact location data for research participants were published in academic journals and newspapers, leading to a breach of spatial confidentiality due to scientists’ or publishers’ negligence [22,23]. To some extent, published maps with masked confidential locations could also be reengineered to reveal the exact location of an individual [22]. Availability of this information in the public domain may increase the risk of identification for the participants. While this is especially pertinent for vulnerable populations, such as people living with HIV, protecting spatial confidentiality and participant identity should be a consideration for GST research in general.

### Challenges in obtaining informed consent

Due to the complex nature of the risks involved in the use of geo-location devices, it may be difficult to ensure that participants are properly informed regarding the possible implications of its use. The problem is likely to be exacerbated in low- and middle-income countries due to lower levels of education and lower availability of GSTs, leading to lack of awareness.

In the care-seeking study, more technical issues such as data encryption and storage and third-party transfer were very difficult to communicate with participants despite good translations of informed consent documents. This was again primarily due to low levels of technology-related awareness among this study population. To overcome these issues, participant information and consent documents for the study were drafted in simple language to explain the maintenance of confidentiality of the study data but did not include complex technical details about data storage and transfer.

### Social and relational dimensions of monitoring

Our experiences in the care-seeking study also emphasize the importance of taking account of the social context in which GPS monitoring interventions are deployed, and the potential impact on social relations, particularly with close family members, of participating in a study involving GST location tracking and unfamiliar devices.

In the case of a few study participants, the smartphones given to the mothers were used by their husbands during their work hours, switching off the GPS tracking facility. Based on feedback from the field research assistants, this was partly due to the husbands’ concern for the safety and confidentiality of their wives, but also due to the fact that they could now get access to a smart phone (which many of them did not have earlier) for their personal use. In fact, it could be argued that availability of smart-phones encouraged participation in the study – though, it should be noted, not in such a way as ought to raise the additional ethical concerns that sometimes arise with respect to incentives and “undue inducements”. There was one reported case of marital dispute during the study period due to the presence of location tracking device with the wife, which led to that family’s non-participation in the study.

Our experience is similar to the one reported by Paz-Soldan *et al* in their study on acceptability of GPS units in Peru [7]. Concerns reported in this study included fears that the device might record their conversations, privacy and confidentiality concerns and safety concerns. The problem of jealousy from partners was also reported in Peru study by some women, as some men might not trust that their wives had been asked to participate in a study and might not believe that the device was for the stated purpose; some suggested that the research team should provide participants with evidence that the GPS unit was part of the study. These experiences suggest a need for better involvement of close family members of the study participants during the consent process and educating them about the use of location tracking technologies in research, as well as caveats.

### Accuracy of data

Geospatial data methods may not always yield accurate information about location. When GST is used in the course of service provision, this leads to questions about liability and responsibility, for example if misleading information leads to financial losses (through relying on incorrect information) or even physical harm (such as in cases where emergency services may be directed to the wrong location).

In the context of research, one concern is that inaccuracy can lead to misinterpretation: for example, a person visiting a shop near a health facility may not be distinguishable from a person sitting inside a health centre. Likewise, a person passing by a health centre or whose place of residence is near a health facility may be inaccurately recorded in the system as having visited the health centre. These events were commonly encountered during the care-seeking study by the field research assistants, but as the timing of each visit was also recorded in the device, this helped the researchers to a large extent to ‘clean’ the captured data. This is one of the limitations of GST that may pose an ethical challenge, but has not been associated with any ethical issue in the context of this care-seeking study. With advances in technology, the accuracy of spatial data continues to improve and hence this problem may be minimized in future [24].

### Unauthorised access to data

Another danger in using GST for research is the possibility of misuse of data through third-party access, for example by internet or Bluetooth hackers, commercial telecommunication agencies or commercial cloud storage providers [2]. There have been documented instances where agencies have gained access to personal data despite data encryption [25]. There is also the potential danger that computer malware or viruses can utilize vulnerabilities of data encryption to gain access to data for commercial or criminal purpose, as for example occurred with the Ransomware scam [26]. Furthermore, security breaches aside, there is a theoretical possibility that the information may be subpoenaed as a part of legal proceedings, in which case the researchers cannot guarantee protection of participants’ privacy [2]. These are some of the loopholes at present as regards data accessibility.

In order to minimize this risk during the care-seeking study, strict implementation of data safety system was observed. This included provision of secured server space, data anonymisation, data encryption, restricted access to collection and storage of care-seeking study movement data generated by smart phones. Nonetheless, these measures have inherent limitations with respect to guarding against potential security breaches or other means by which data may be accessed. The need to address ethical issues posed by the risk of data security breach or misuse, however, goes well beyond the sphere of research; rather than being a matter for the governance of individual research projects, it will require broader societal consideration as to the possible harms of data misuse and how, when absolute security cannot be guaranteed, these harms should be compensated or protected against.

## CONCLUSIONS

GPS-based movement tracking presents a classic example of the ‘double-edged sword’ with respect to its use in health research, given that the detailed information it can reveal is the source of both its value for research and the ethical concerns it presents. The ultimate challenge lies in permitting legitimate use without allowing misuses. Anonymisation of study participants, de-identification of unnecessary location data, explanation of possible ethical issues and available solutions in simple language during informed consent, selective archival of study-related geospatial data and secure storage of data are specific measures that were taken during care-seeking study in order to address potential ethical concerns. Difficulty in explaining the complex nature of ethical implications to participants in rural areas, and the potential danger of security breaches remain as limitations.
